# Long-term activity drives dendritic branch elaboration of a *C. elegans* sensory neuron

**DOI:** 10.1016/j.ydbio.2020.01.005

**Published:** 2020-05-01

**Authors:** Jesse A. Cohn, Elizabeth R. Cebul, Giulio Valperga, Lotti Brose, Mario de Bono, Maxwell G. Heiman, Jonathan T. Pierce

**Affiliations:** a Institute for Cellular and Molecular Biology, Department of Neuroscience, The University of Texas at Austin, TX, USA; b Department of Genetics, Blavatnik Institute, Harvard Medical School and Boston Children’s Hospital, Boston, MA, USA; c Division of Cell Biology, Medical Research Council Laboratory of Molecular Biology, Cambridge, United Kingdom

## Abstract

Neuronal activity often leads to alterations in gene expression and cellular architecture. The nematode *Caenorhabditis elegans*, owing to its compact translucent nervous system, is a powerful system in which to study conserved aspects of the development and plasticity of neuronal morphology. Here we focus on one pair of sensory neurons, termed URX, which the worm uses to sense and avoid high levels of environmental oxygen. Previous studies have reported that the URX neuron pair has variable branched endings at its dendritic sensory tip. By controlling oxygen levels and analyzing mutants, we found that these microtubule-rich branched endings grow over time as a consequence of neuronal activity in adulthood. We also find that the growth of these branches correlates with an increase in cellular sensitivity to particular ranges of oxygen that is observable in the behavior of older worms. Given the strengths of *C. elegans* as a model organism, URX may serve as a potent system for uncovering genes and mechanisms involved in activity-dependent morphological changes in neurons and possible adaptive changes in the aging nervous system.

## Highlights

• The dendritic tip of an oxygen-sensing neuron grows elaborate microtubule-rich processes in adult *C. elegans*.
• Dendritic tip elaboration depends on the long-term activity of the neuron and calcium.
• The elaboration correlates with increased sensitivity of the neuron to certain ranges of oxygen as well as higher avoidance of oxygen during bordering behavior.
• The dendritic tip changes may reflect adaptive changes in physiology and behavior during adulthood.

## 1 Introduction

The nervous system often displays morphological plasticity in response to prolonged input or activity. These activity-dependent changes in neuron shape allow animals to interact more adeptly with their environment. For instance, the growth and pruning of specific synapses as well as axon and dendritic branches allow neural circuits to alter synaptic weighting during forms of learning and homeostatic plasticity (Yang et al., 2014; Yuste and Bonhoeffer, 2001). Interneurons also adjust the number and shape of their minute dendritic spines to filter input differently in neuronal networks (Katzel and Miesenbock, 2014; Huang et al., 2016; Lee et al., 2008). In the sensory system, photoreceptor outer segment length has been shown to change in response to different light levels (Abramoff et al., 2013). Thus, although the gross structure of the adult nervous system often remains static, many neurons change shape at subtle spatial and temporal scales.

The transparency, genetic tractability, and compact nervous system of the nematode *C. elegans* make the worm an excellent system to study genes that underlie how neurons achieve and adjust their shape. Many aspects of neuronal morphology have been examined in *C. elegans*, such as axonal and dendritic establishment (Chisholm et al., 2016; Kirszenblat et al., 2011), dendritic tiling (Yip and Heiman, 2016), synapse specification (Seifert et al., 2006), and sensory cilia morphogenesis and maintenance (Inglis et al., 2007; Howell and Hobert, 2017). The worm has also been used to study how neurons alter their shape in response to changes in environment, such as the reshaping of the ciliated chemosensory neuron AWB by sensory activity (Mukhopadhyay et al., 2008), and the restructuring of sensory neuronal endings in an alternative developmental larval stage termed dauer, which is induced by certain environmental conditions (Procko et al., 2011; Schroeder et al., 2013; Albert and Riddle, 1983). Furthermore, many of the genes required for the development and maintenance of sensory cilia in *C. elegans* have conserved roles across species (Ansley et al., 2003; Ou et al., 2007).

Most sensory neurons in the head of *C. elegans* are bilaterally symmetric and have a cell body that projects a single dendrite to the tip of the nose, where the sensory transduction machinery is often localized (White et al., 1986). Here we focus on one class of these sensory neurons, the oxygen sensing neuron pair URX. In its natural environment where it burrows through rotting vegetation, *C. elegans* experiences a wide range of oxygen levels from nearly anaerobic patches (1% oxygen) to surface level oxygen (21%) (Scott, 2011). When assayed in an oxygen gradient in the lab, worms exhibit a preference for 7–10% oxygen environments, which reflects their preferred oxygen concentrations in the wild (Zimmer et al., 2009; Gray et al., 2004). This migratory behavior, termed aerotaxis, is primarily driven by the URX neurons. Mutant worms that lack components of the oxygen sensory transduction pathway in URX or worms that have URX ablated are deficient in aerotaxis (Chang and Bargmann, 2008; Cheung et al., 2004). Calcium imaging experiments have revealed that, unlike many other sensory neurons in worm that respond phasically to changes in stimuli, URX neurons remain tonically active at ambient oxygen (21%) (Busch et al., 2012).

We report here that continuous exposure to surface level oxygen causes the URX neuron to steadily grow elaborate branches at its dendritic sensory ending over the course of adulthood. Branch elaboration depends on oxygen levels because cultivating worms in low oxygen (1% O_2_) prevented growth of these complex-shaped dendritic tips. We also find that the oxygen sensory pathway is necessary for this growth, suggesting that branch elaboration is due to neuronal activity. The components of the oxygen sensing pathway normally localize to a position at the end of the dendrite just beneath the surface of the nose of the worm, where they are thought to assemble into a signaling microdomain (Gross et al., 2014; Abergel et al., 2016). Using a fluorescent tag, we found that the activity-dependent dendritic branches in most worms do not contain GCY-35, a guanylyl-cyclase necessary for oxygen sensation (Gray et al., 2004), and so do not appear to extend the oxygen signaling compartment for this molecular sensor.

We also investigated whether the change in URX morphology in day four adults corresponded with a change in physiology. We tested this at the cellular level by imaging a genetically encoded calcium sensor while exposing worms to different oxygen levels, and at the behavioral level by assaying an oxygen-dependent behavior called “bordering” (Gray et al., 2004). We found that URX in older worms had an increased sensitivity to certain ranges of oxygen, and that this also correlated with an increased aversion to ambient oxygen at the behavioral level. Thus, URX appears to show a change in acuity as the worm ages that correlates with the morphological changes that occur at the dendritic ending.

Sensory endings in *C. elegans* have been compared to several different structures in higher animals, such as dendritic spines, sensory cilia, and primary cilia (Mukhopadhyay et al., 2008; Shaham, 2010). In addition, many of the components of the URX sensory cascade have homologous counterparts in the nervous systems of higher animals. URX may therefore serve as a powerful system for identifying important conserved genes and mechanisms involved in activity-driven morphological changes in other species, and as an example of neuronal sensitivity changing with age.

## 2 Results

### 2.1 Oxygen sensation drives dendritic branch elaboration in the oxygen-sensing neuron pair URX

**Fig. 1:**
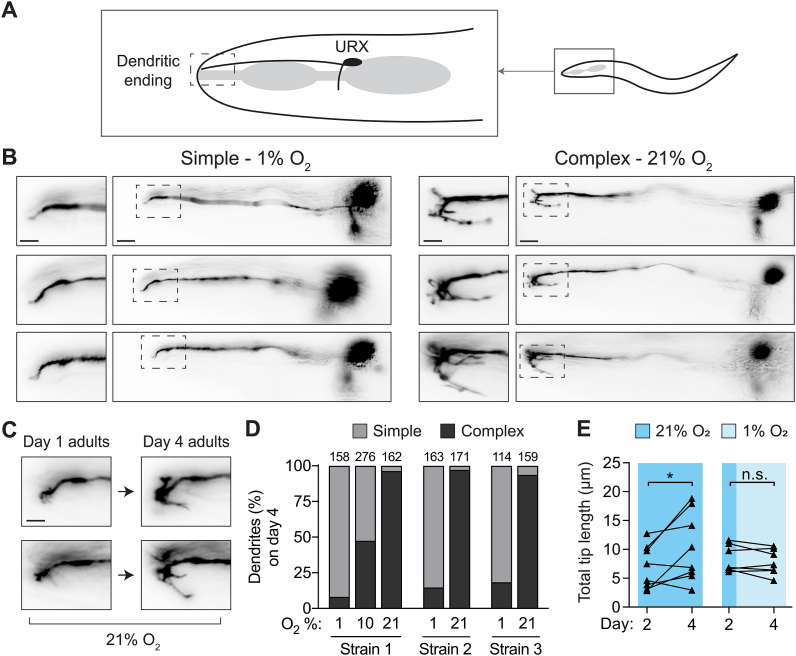
**Environmental oxygen drives branching of the dendritic endings of the URX neuron pair** A.) Cartoon representation of the locations of the URX cell body and dendritic ending in the head of the worm. In all images, anterior is to the left, dorsal is upwards. B.) Examples of simple and complex dendritic ending morphologies in URX in wild-type worms. URX was visualized by expressing a *Pgcy-32::GFP* transgene, and worms were grown in either high (21%) or low (1%) oxygen until day four of adulthood. Note variable branched morphology of complex dendritic endings. Scale bars in images showing full neuron and inset are 10 ​μm and 5 ​μm, respectively. C.) The same individuals were imaged on day one and day four of adulthood to show the growth of branches over time in 21% oxygen. Scale bar is 5 ​μm. D.) Scoring of dendritic ending morphology in wild-type worms grown and maintained at either 21%, 10%, or 1% oxygen until day four of adulthood. Three independently-derived strains were scored. In this and all similar figures, the number of dendrites scored per condition is shown above each bar. E.) Wild-type worms were reared in high oxygen until day two of adulthood, and then maintained in either high or low oxygen until day four of adulthood, with images taken at both time points. Branched endings in worms maintained at high oxygen showed significant growth over these two days (t ​= ​2.5, p ​< ​0.05), while those in low oxygen neither grew nor shrank appreciably (t ​= ​1.2, p ​> ​0.05). n ​= ​8 for high oxygen conditions, 7 for low oxygen condition. Statistical significance determined by paired *t*-test.

*C. elegans* uses the bilaterally symmetric neuron pair URX to sense environmental oxygen levels (Zimmer et al., 2009; Gray et al., 2004; Busch et al., 2012). The URX cell body is located in the head of the worm near the posterior pharyngeal bulb. Each URX neuron extends an axon into the nerve ring and a dendritic process to the nose of the worm where it is anchored via connections with the ILso glial cell (Fig. 1A) (White et al., 1986; Doroquez et al., 2014; Ward et al., 1975; Cebul et al., 2019). This dendritic process ends just beneath the skin, where environmental oxygen may diffuse a short distance to bind the molecular receptor for oxygen, a class of membrane-anchored guanylyl-cyclases (*gcy*) (Gross et al., 2014). Previous studies of the *C. elegans* nervous system describe the URX dendritic tip as having branched endings that are variable in size and morphology (White et al., 1986; Doroquez et al., 2014; Ward et al., 1975). While investigating gene expression in URX using fluorescence microscopy, we noticed that worms grown in low oxygen environments (1% O_2_) fail to sprout branches at the ends of the URX dendrites (Fig. 1B left), in contrast to the branches seen in worms grown in high oxygen (21% O_2_) (Fig. 1B right).

To study the effect of oxygen level on branch elaboration, we visualized URX neurons using cytoplasmic GFP driven by the *gcy-32* promoter. This *gcy-32* reporter is robustly expressed in URX, AQR, and PQR neurons (Yu et al., 1997), but because neither AQR nor PQR send processes to the nose, we could clearly visualize the dendritic endings of URX in these strains. We characterized the dendritic morphology of URX in three independently-derived transgenic strains to control for artifacts caused by variation in GFP expression (Kadandale et al., 2009).

We imaged individual worms repeatedly across each day of adulthood and observed that URX dendritic branches in worms maintained at high oxygen levels continued to grow in length and complexity as the animal aged (Fig. 1C). By day four of adulthood, the difference between the dendritic branches of worms grown in 21% and those grown in 1% was pronounced, so we chose this particular age to quantify differences between conditions. The morphological variability of the dendritic tips was difficult to describe; however, we found that we could unambiguously classify dendritic tips with elaborate branches as “complex”, and those without as “simple”. Specifically, if the dendritic tip had at least one secondary branch longer than 5 ​μm, we classified it as complex; otherwise the dendritic tip was classified as simple. In worms grown in 21% oxygen, the vast majority of dendritic tips were complex in each of the three transgenic reporter lines (complex ​= ​96.3%, 97.1%, and 93.7%), while worms grown in 1% oxygen had mostly simple dendritic tips (complex ​= ​8.2%, 14.7%, 18.4%). We also examined URX endings at 10% oxygen, and found they had an intermediate phenotype (complex ​= ​47.5%) (Fig. 1D). These results show that oxygen drives growth of elaborate branches at the end of the URX sensory dendrite, and that growth continues as long as the worm remains exposed to oxygen.

### 2.2 Dendritic branches in URX are not actively broken down in low oxygen

The sensory transduction proteins in URX are localized to the dendritic ending (Gross et al., 2014), so we hypothesized that the growth of the complex branching might represent a form of homeostatic plasticity where the tips would expand in high oxygen and be broken down in low oxygen in order to regulate the amount of sensory receptors in the dendritic ending. To test this idea, we raised worms at 21% oxygen until day two of adulthood, at which point we quantified the total length of the branches on a single dendritic tip per worm. The imaged worms were then individually recovered and maintained for the next 2 ​days ​at either high or low oxygen, at which point we again quantified the total length of the branches (Fig. 1E). We found that while the dendritic branches in worms kept in high oxygen (21%) continued to grow over the two days, the branches in worms moved to low oxygen (1%) neither grew nor reduced, but rather stayed the same length. Thus, dendritic branches in URX are not broken down in a low oxygen environment once established, and a visible “imprint” of high oxygen exposure remains encoded in the morphology of the URX ending.

### 2.3 The oxygen sensing pathway is necessary for dendritic branch growth in URX

**Fig. 2:**
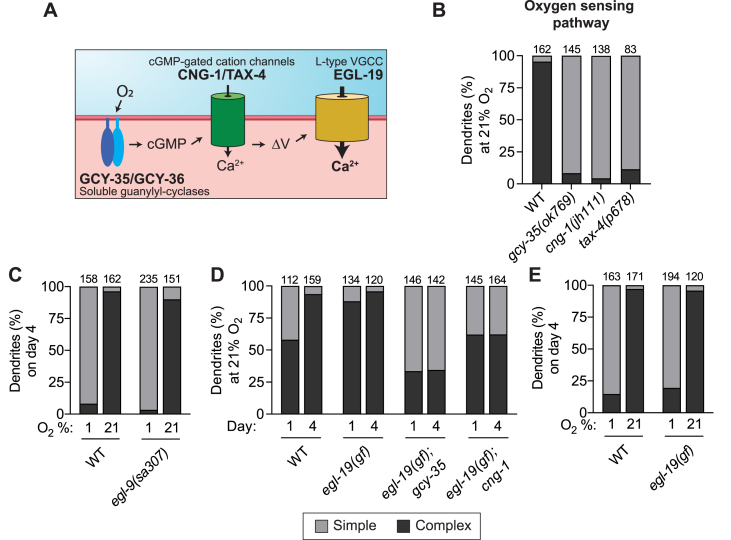
**Sensory activity is necessary for elaboration of branched endings in URX** A.) Schematic showing the oxygen-sensing pathway in URX. A dendritically-localized heterodimer of GCY-35/GCY-36 produces cGMP after binding oxygen. cGMP then activates downstream CNG cation channels CNG-1 and TAX-4. Membrane depolarization subsequently gates the L-type voltage-gated calcium channel EGL-19. B -E.) URX dendritic ending morphology was scored in worms of the given genotype and age after growth in the indicated oxygen concentration. Components of the oxygen-sensing pathway were necessary for dendritic branch elaboration, while stabilization of the hypoxia-induced transcription factor HIF-1 in the *egl-9(sa307)* mutant had no effect on branch growth. The gain-of-function *(gf)* allele *egl-19(n2368)* did not drive branching in worms grown at 1% oxygen, but it was sufficient to significantly increase branching in *gcy-35* and *cng-1* mutant backgrounds. Each bar is representative of three independently-derived transgenic strains expressing *Pgcy-32::GFP* to visualize URX.

URX is a sensory neuron for oxygen, which suggests the possibility that branch growth at the dendritic tip is caused by prolonged sensory activity. In URX, molecular oxygen is coordinated at the dendritic tip by a heterodimer of the membrane-tethered guanylyl cyclases GCY-35 and GCY-36 (Gray et al., 2004; Gross et al., 2014; Abergel et al., 2016). Together these proteins produce intracellular cGMP when bound to oxygen, which in turn activates the cyclic nucleotide-gated cation channels TAX-4 and CNG-1 (Gray et al., 2004; Cheung et al., 2004; Couto et al., 2013) (Fig. 2A). We examined mutants lacking *gcy-35, cng-1,* or *tax-4*, and found that each mutant was profoundly defective in growing branches at the dendritic tip in URX when maintained at 21% oxygen until day four of adulthood (complex ​= ​8.3%, 4.4%, 6.5%, respectively) (Fig. 2B). This strongly suggests that oxygen sensation drives branch elaboration at the URX dendritic tip.

We also considered the alternative hypothesis that branch growth is repressed by the hypoxia pathway in low oxygen (1%) and thus is revealed at high oxygen (21%). To test this hypothesis, we examined mutants lacking the prolyl hydroxylase EGL-9, which mediates degradation of the hypoxia pathway transcription factor HIF-1 under high oxygen conditions (Jiang et al., 2001). In *egl-9* mutants, the hypoxia pathway is constitutively active (Epstein et al., 2001). We found that wild-type and *egl-9* mutant worms had similar URX branching at 21% oxygen (complex in *egl-9* ​= ​90.1%) and at 1% oxygen (complex in *egl-9* ​= ​3.4%) (Fig. 2C), indicating that HIF-1-dependent gene expression is likely not involved in repressing dendritic branch growth in low oxygen conditions.

Taken together, these results support the idea that the GCY-35/GCY-36-CNG-1-TAX-4 oxygen sensing pathway drives branch growth under high oxygen conditions, and that the lack of branch growth in low oxygen conditions is due to decreased activity of URX, not repression by the hypoxia pathway.

### 2.4 Increasing intracellular calcium is sufficient to drive dendritic branch growth

Both cGMP and calcium levels increase in URX as a result of oxygen sensation. To determine if calcium alone is sufficient for dendritic branch growth, we took advantage of a gain-of-function allele of *egl-19*, the sole L-type voltage-gated calcium channel in *C. elegans* (Laine et al., 2014). The EGL-19 calcium channel is gated after depolarization of the URX neuron by CNG-1 and TAX-4 channels (Fig. 2A). The *egl-19(n2368)* allele encodes an EGL-19 channel that opens at more hyperpolarized membrane potentials and has a slower inactivation compared to the wild-type protein. This gain-of-function (*gf*) mutant background allowed us to test whether increased intracellular calcium influx in URX could induce branch growth in wild-type at 21% and 1% oxygen and the oxygen sensory transduction mutant backgrounds *gcy-35* and *cng-1* at 21% oxygen (Fig. 2D and E).

First, we found that the *egl-19(gf)* allele in an otherwise wild-type background caused precocious branch elaboration in URX, with 88% of dendritic endings in day one adult worms showing complex branches, compared to only 58% in wild-type worms. This result suggests that extra calcium via EGL-19 may be sufficient to promote URX branch elaboration. Second, we found that crossing the *egl-19(gf)* mutation to the *gcy-35* or *cng-1* oxygen sensory transduction mutants dramatically increased their proportion of complex URX branches. We found that the URX dendritic ending in the *egl-19(gf);gcy-35* double mutant showed 35% complex branches, compared to 8.3% in the *gcy-35* single mutant. In the *egl-19(gf);cng-1* double mutant, 65% of URX dendritic endings had complex branches, compared to 4.4% in the *cng-1* single mutant (Fig. 2D). Intriguingly, the URX dendritic ending in the *egl-19(gf);cng-1* double mutant often took on a blobby appearance when compared with other backgrounds, examples of which are shown in Supplemental Fig. 1. We also found that *egl-19(gf)* mutant worms reared at 1% oxygen until day four of adulthood failed to grow more elaborated URX endings than wild-type worms (Fig. 2E). This could be because the *gcy-35* and *cng-1* mutants are not completely analogous to worms grown in 1% oxygen, or because residual cGMP and calcium in URX in these mutant backgrounds at 21% oxygen is sufficient to open the more sensitive EGL-19 channel.

Taken together, the above results suggest that increased intracellular calcium influx via the EGL-19 ​L-type calcium channel is sufficient to stimulate elaboration of URX dendritic branches at 21% oxygen even in the absence of components of the oxygen sensation cascade.

### 2.5 Wild-type URX dendrites have exuberant branching and an intricate internal structure

**Fig. 3:**
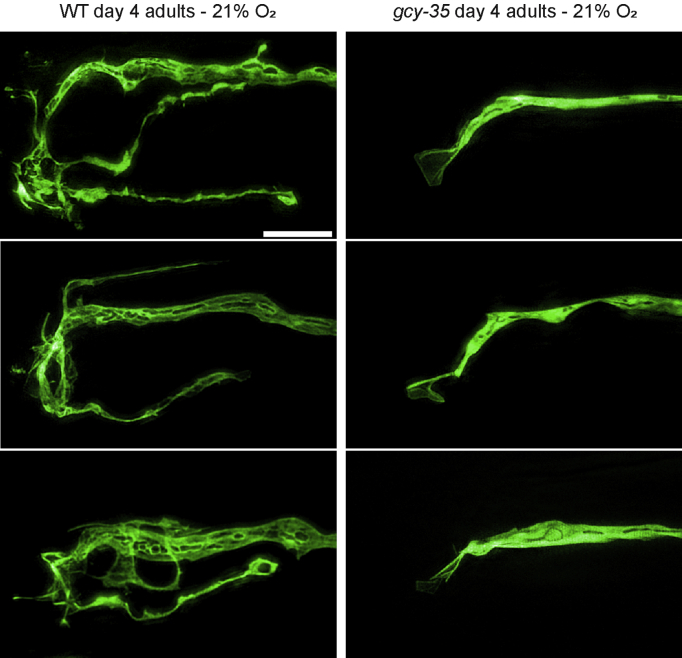
**High-resolution images of branched URX dendritic endings.** Representative high-resolution images of the dendritic endings of URX in either wild-type or *gcy-35(ok769)* mutant worms. All animals were grown in 21% oxygen until day four of adulthood. Scale bar is 5 ​μm.

We next used super-resolution microscopy to gain a more detailed look at the dendritic branches or lack thereof in wild-type and *gcy-35(ok769)* day four adult worms (Fig. 3). These higher resolution pictures of URX confirm the complex morphology of dendritic endings in wild-type worms and the lack of branched dendritic endings in the *gcy-35* mutant. In addition, we noticed several interesting features at this resolution that we were unable to discern by conventional microscopy. For one, nearly all *gcy-35* mutant individuals had a small membranous fan structure attached to the end of the dendritic stalk. This is reminiscent of the membranous fan-like structures that were observed in the dendritic tips of the AWB chemosensory neurons in similar sensory transduction mutants (Mukhopadhyay et al., 2008; Doroquez et al., 2014; Ward et al., 1975). We also noticed several areas in the dendritic stalk and in the outgrown branches that excluded GFP, leading to an intricate and complicated internal structure. We hypothesize that these exclusions may be secretory vesicles, and could thus reflect dense secretory traffic in the wild-type dendrite to facilitate branching at the dendritic ending, or they may be other membranous organelles such as endosomes.

### 2.6 Branched URX dendritic endings contain microtubules and do not appear to expand an oxygen sensory compartment

To learn more about the nature of the branched dendritic endings in URX, we utilized a strain in which the microtubule-binding protein EBP-2 is tagged with GFP in order to determine whether the cellular cytoskeleton extends into the outgrown dendritic branches (Harterink et al., 2018). In day four adult worms, we found that EBP-2:GFP was present throughout URX, including the dendritic branches, in all individuals examined (n ​= ​15/15). This suggests that microtubules may play a role in cytoskeletal support and/or cellular transport in these branched dendritic endings.

**Fig. 4:**
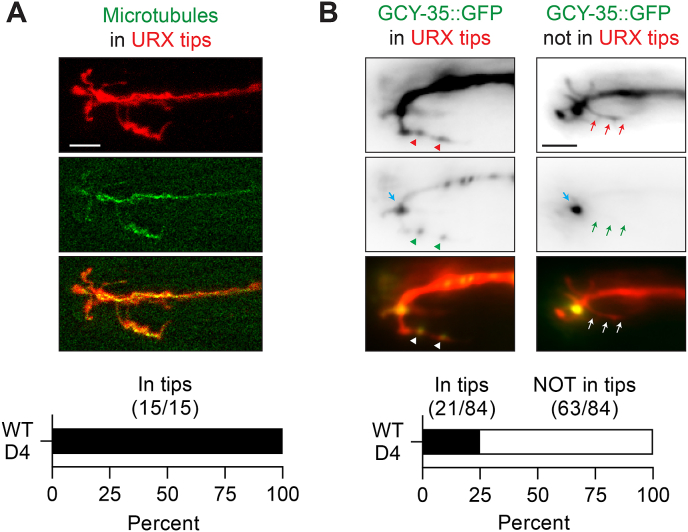
**Branched URX dendritic endings contain microtubules and are likely not an expansion of an oxygen sensory compartment** A.) An example day four wild-type adult grown in high oxygen and expressing soluble mCherry cell-specifically to label URX, and EBP-2: GFP in URX to label microtubules. Scale bar is 5 ​μm. B.) Localization of GCY-35: GFP in URX in day four wild-type adults grown in high oxygen. Blue arrows label a likely sensory compartment in URX where GCY-35::GFP is highly concentrated in all animals. The red arrows and arrowheads point to an outgrown branch of URX, and green arrows/arrowheads point to either visible GFP, or lack thereof in the branches. 25% of animals examined had visible GFP signal in the outgrown dendritic branches (left), while 75% did not (right). Scale bar is 5 ​μm.

Components of the oxygen-sensing machinery, including the guanylyl cyclases GCY-35 and GCY-36, localize to the ending of the dendritic stalk in URX, where they are thought to associate with one another to form a signaling microdomain (Gross et al., 2014; Abergel et al., 2016). We used a strain in which GCY-35 is tagged with GFP to investigate whether the dendritic branches in day four adults contain components of the oxygen sensing pathway and may therefore be an expansion of the URX oxygen sensory compartment (Fig. 4B). We found that in all worms examined, GCY-35:GFP localized to the end of the dendritic stalk in the nose, and in 75% of worms (n ​= ​63/84), GCY-35:GFP was not visible in the outgrown dendritic branches. In the remaining 25% of worms (n ​= ​21/84), we noticed GFP signal in the outgrown branches, though usually at a much lower level than is seen at the end of the dendritic stalk. Although it is possible that GCY-35:GFP is present at levels below our limit of detection, or untagged GCY-35 is present in the branches, we interpret these data to mean that the outgrown branches at the dendritic ending of URX are likely not an expansion of the sensory compartment.

### 2.7 Dendritic branches correlate with an increase in sensitivity to certain ranges of oxygen in older animals

URX responds to oxygen tonically. Ambient environmental oxygen causes a proportional steady intracellular calcium influx into URX (Busch et al., 2012). This in turn causes URX to signal downstream interneurons and thereby drive oxygen-related behaviors. Because some sensory neurons have distinct spatial compartments that contribute to different aspects of their function (Nguyen et al., 2014), we hypothesized that the complex branches at the URX dendritic tip might enhance oxygen sensing. If so, enhanced oxygen sensation may be reflected by alterations in cellular calcium responses to oxygen or in oxygen-related behaviors. To test this hypothesis, we compared the cellular and behavioral responses to oxygen in wild type day one adults and day four adults grown in 21% oxygen, because day four adults have more elaborated and developed branches at the URX dendritic ending than day one adults. We chose not to study the cellular and behavioral responses to oxygen for the sensory mutants described above because although they fail to grow complex branches, they are impaired in oxygen sensation. Likewise, we did not study worms maintained in 1% oxygen because although this prevents complex branching, it may also cause the neuron to adapt in a way that would confound our results (Gross et al., 2014).

**Fig. 5:**
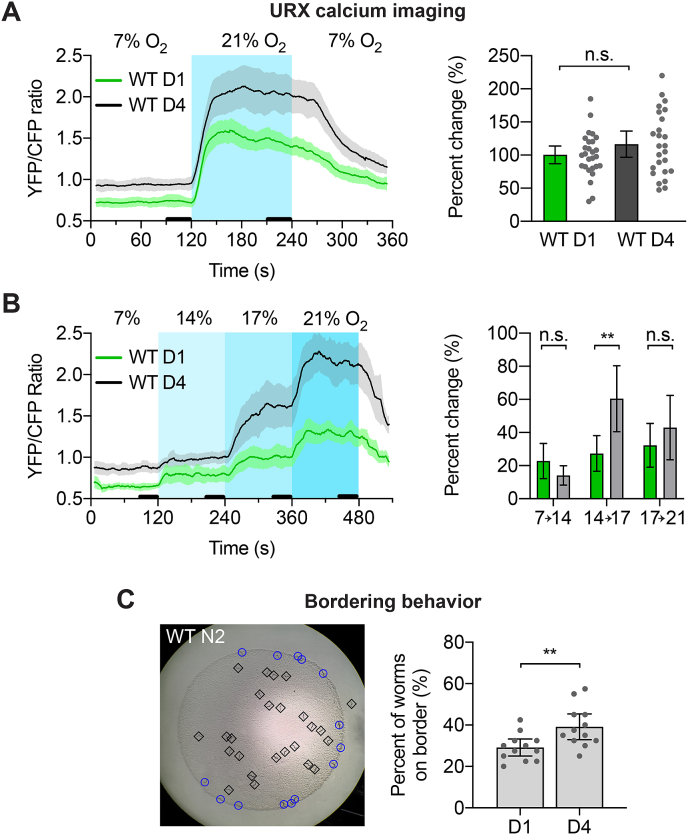
**URX dendritic ending branch length/complexity correlates with an increased sensitivity to certain ranges of oxygen** A.) Wild-type URX calcium responses to 7%–21% - 7% oxygen steps. Older worms had higher intracellular calcium at both oxygen levels, but no significant difference in the percent change in calcium upon the oxygen shift. Shaded areas on the calcium trace are 95% confidence intervals. Thick black lines near x-axis show the time intervals used to calculate percent change. Error bars on percent change are 95% C.I. N ​= ​27 for day one adults, 25 for day four adults. p ​= ​0.16 as determined by Student’s t-test. B.) Same as in A, but with oxygen shifts from 7% to 14% to 17% to 21% oxygen. Older worms again had higher intracellular calcium at all oxygen levels, and had a significantly larger response to the 14%–17% percent shift. N ​= ​23 for day one adults, 24 for day four adults. p-values for 7%–14%, 14%–17%, 17%–21%, adjusted for multiple t-tests: 0.42, 0.01, 0.99. C.) Bordering assays for wild-type day one and day four adults. Image on the left shows an example of a bordering assay. Day four adults were found on the border significantly more than day one adults, indicating an altered oxygen preference. Each point is an assay of 40 worms. N ​= ​12 assays for day one adults, 12 for day four adults. p ​< ​0.01 as determined by Student’s t-test.

To compare the cellular response to oxygen, we used the ratiometric calcium indicator yellow cameleon 2.60 (YC2.60) to measure calcium levels in the cell body of URX in response to step changes in environmental oxygen (Nagai et al., 2004). We first tested a shift from 7% O_2_ to 21% O_2_ and back to 7% O_2_. We found that day four adults had higher levels of calcium at both 7% and 21% oxygen compared to day one adults, and that the percent change in calcium upon shifting to higher oxygen was similar at both ages (Fig. 5A).

We next tested a more nuanced paradigm that used smaller steps from 7% to 14% to 17% to 21% oxygen. These smaller shifts likely more closely approximate the gradual changes a worm would typically encounter while moving through their environment. As before, we found that intracellular URX calcium in day four adults was higher at all oxygen levels compared to day one adults, and both ages had similar changes in calcium in response to 7% to 14% and 17% to 21% oxygen shifts. However, the shift from 14% to 17% oxygen elicited a significantly larger change in calcium in day four adults compared to day one adults (Fig. 5B). This suggests that the sensitivity of URX for particular ranges of oxygen changes as worms age.

We also examined a URX-dependent behavior called bordering. Wild isolates of *C. elegans* prefer conditions of 5–12% environmental O_2_ and avoid higher levels of oxygen (Gray et al., 2004). Consequently, wild isolate worms in the lab will accumulate at the border of a bacterial lawn where the bacteria grows thickest and local oxygen levels are closest to their preferred level. Worms lacking URX or components of the oxygen sensory pathway no longer display this bordering behavior (Zimmer et al., 2009; Gray et al., 2004). A gain-of-function mutation in the gene *npr-1* in the standard lab strain N2 causes them to have a blunted oxygen aversion compared to wild isolates, and therefore N2 worms also have a less pronounced bordering behavior (Gray et al., 2004; de Bono and Bargmann, 1998).

The bordering assay is performed by placing worms onto an agar plate seeded with an *E. coli* food patch, letting the worms move freely around the plate for 1 ​h, and then quantifying how many worms are on the border of the bacterial lawn (Fig. 5C). During this assay worms encounter 21% oxygen while off of the food lawn, 17% oxygen in the center of the food lawn, and 13% oxygen while in the thickened border of the food lawn (Gray et al., 2004). The transition from the food border to the center of the food roughly corresponds to the 14%–17% shift in our imaging assays. We found that wild type day 4 adults had a subtle but significant increase in bordering compared to day one adults (Fig. 5C). We also tested *npr-1(ky13)* mutant worms, and saw that day four worms displayed less bordering than day one adults, though *npr-1* mutant worms have altered oxygen circuit dynamics compared to wild-type N2 worms, so this discrepancy could be due to several factors (Supplemental Fig. 2) (Chang et al., 2006). Nevertheless, the change in URX sensitivity we saw at the cellular level in older wild-type worms corresponds with a change in the URX-driven bordering behavior, and correlates with the outgrowth of the dendritic tips in URX described above.

## 3 Discussion

### 3.1 Morphological changes in *C. elegans* sensory neurons

*C. elegans* is a powerful system for studying changes in neuron morphology, as the transparency of the animal allows for convenient imaging of fluorescently labeled, identified cells in live animals across different ages and conditions. The *C. elegans* literature has several examples of developmental changes in neuronal shape, such as the pruning of excessive neurites (Kage et al., 2005), and the restructuring of certain sensory neurons during the dauer alternate larval stage (Procko et al., 2011; Schroeder et al., 2013; Albert and Riddle, 1983). There are also a few examples in adulthood, such as the age-dependent increase in the branching of the PVD mechanosensory neurons (Smith et al., 2010), and the abnormal morphology of some sensory neuron endings in signal transduction mutant backgrounds (Singhvi et al., 2016; Roayaie et al., 1998). However, to our knowledge, there is only one other clear example of environmental input directly influencing sensory ending morphology in *C. elegans*, which is that of the AWB chemosensory neurons (Mukhopadhyay et al., 2008). The AWB sensory dendritic ending has been shown to remodel from two finger-like ciliated branches to two fan-like structures in the absence of olfactory sensory input. This change was dependent on sensory transduction genes and the kinesin-II motor protein, among other factors. Interestingly, several other neurons in *C. elegans* with characteristic morphologies, such as AFD, do not show obvious remodeling of their sensory ending depending on neuronal activity (Singhvi et al., 2016). Why some sensory neurons in *C. elegans* undergo shape changes in response to activity while others do not is an intriguing question for further analysis.

Recently, McLachlan et al. described a role for the *C. elegans* MAPK15/ERK8 homologue in controlling dendritic length in URX (McLachlan et al., 2018). In the *mapk-15* mutant, URX grows extremely long dendritic endings, much longer than those we describe here. Despite these long dendritic overgrowths, the *mapk-15* mutant displayed a roughly normal response to oxygen at both cellular and behavioral levels. One interesting difference between the dendritic overgrowths in the *mapk-15* mutant and the shorter branches we see in wild-type URX neurons is the localization of the GCY-35 guanylyl-cyclase. In the *mapk-15* mutant, GFP-tagged GCY-35 was observed throughout the dendritic overgrowths, whereas we did not see GCY-35:GFP in the complex dendritic branches of most wild-type worms. One explanation for this discrepancy might be that MAPK-15 defines and constrains the size of an oxygen-sensing compartment at the end of the URX sensory dendrite. In the *mapk-15* mutant then, this sensory compartment is expanded, whereas in wild type, the complex dendritic outgrowths we see represent a distinct subcellular compartment. Though this idea is speculative, sensory ending compartmentalization has been reported in other *C. elegans* neurons (Nguyen et al., 2014).

### 3.2 URX dendritic branches correlate with a change in oxygen sensitivity in older worms

We found that older worms had higher intracellular calcium in URX at all oxygen levels tested, a heightened response to shifts from 14% to 17% oxygen, and an increased propensity to reside at the border of the bacterial lawn on a seeded agar plate. Together these findings point to a physiological change in URX that occurs as the worm ages, which correlates with the increase in branch elaboration at the dendritic tip.

Multiple interpretations could explain our data. For example, because URX calcium levels were higher at all oxygen levels in day four worms, it could be that the overall oxygen preference is shifted lower in day four worms, which would lead to increased bordering. Alternatively, the increased response to shifts from 14% to 17% might cause day four adults to leave the border less often than day one adults. Further work will be needed to determine the nature of the change in URX as the worm ages. Previous work has already shown that certain neuroglobins are able to “tune” aspects of the URX oxygen response (Oda et al., 2017), so it will be interesting to find the mechanism by which URX sensitivity in the 14%–17% range changes with aging.

While we show that the change in URX physiology correlates with its change in morphology, more work will be needed to address whether and how these two phenomena are actually related. The elongation of the dendritic ending may change membrane capacitance and cellular volume, or they may house additional sensory proteins used for oxygen sensation other than GCY-35, among other possibilities. These differences could feasibly contribute to the change in URX physiology, though future experiments will be needed to address these ideas more concretely.

In summary, we have shown that the URX sensory neurons in *C. elegans* grow an elaborate branched structure at their dendritic endings in an activity-dependent manner, and that this morphological change correlates with a change in the sensory responses of URX in older worms. In all animals, the ability for neurons to alter aspects of their shape and physiology is central to their proper functioning. *C. elegans* has contributed much to our understanding of the genes and rules involved in the organization, morphogenesis, and function of the nervous system in mammals. URX may therefore serve as a powerful system for identifying genes important in activity-dependent shape modifications in neurons in higher animals, and for studying how sensory neurons change with age.

## 4 Materials and methods

### 4.1 Strains

#### 4.1.1 The following strains were used

N2 Bristol; JPS879 *vxEx879[Pgcy-32*:*GFP Punc-122::GFP]*; JPS880 *vxEx880[Pgcy-32::GFP Punc-122::GFP]*; JPS881 *vxEx881[Pgcy-32::GFP Punc-122::GFP]*; JPS882 *gcy-35(ok769) I; vxEx882[Pgcy-32::GFP Punc-122::GFP]*; JPS883 *gcy-35(ok769) I; vxEx883[Pgcy-32::GFP Punc-122::GFP]*; JPS884 *gcy-35(ok769) I; vxEx884[Pgcy-32::GFP Punc-122::GFP]*; JPS941 *cng-1(jh111) V; vxEx941[Pgcy-32::GFP Punc-122::GFP]*; JPS942 *cng-1(jh111) V; vxEx942[Pgcy-32::GFP Punc-122::GFP]*; JPS943 *cng-1(jh111) V; vxEx943[Pgcy-32::GFP Punc-122::GFP]*; JPS921 *tax-4(p678) III; vxEx921[Pgcy-32::GFP Punc-122::GFP]*; JPS922 *tax-4(p678) III; vxEx922[Pgcy-32::GFP Punc-122::GFP]*; JPS923 *tax-4(p678) III; vxEx923[Pgcy-32::GFP Punc-122::GFP]*; JPS1103 *egl-9(sa307) V; vxEx1103[Pgcy-32::GFP Punc-122::GFP]*; JPS1104 *egl-9(sa307) V; vxEx1104[Pgcy-32::GFP Punc-122::GFP]*; JPS1105 *egl-9(sa307) V; vxEx1105[Pgcy-32::GFP Punc-122::GFP]*; JPS1079 *egl-19(n2368) IV; vxEx1079[Pgcy-32::GFP Punc-122::GFP]*; JPS1159 *gcy-35(ok769) I; egl-19(n2368) IV; vxEx1079[Pgcy-32::GFP Punc-122::GFP]*; JPS1160 *egl-19(n2368) IV; cng-1(jh111) V; vxEx1079[Pgcy-32::GFP Punc-122::GFP]*; JPS1161 *hrtSi4[Pgcy-36*:*EBP-2::GFP]; vxEx1161[Pgcy-32::mCherry ​+ ​rol-6(su1006)]*; JPS1123 *dbEx[Pgcy-37::GCY-35::HA::GFP::SL2::mCherry]*; AX7629 *dbEx[Pgcy-37::YC2.60::unc-54UTR Punc-122::RFP]*; CX4148 *npr-1(ky13) X*; JPS1173 *npr-1(ky13) X; vxEx1173[Pgcy-32::GFP Punc-122::GFP]*; JPS1174 *npr-1(ky13) X; vxEx1174[Pgcy-32::GFP Punc-122::GFP]*; JPS1175 *npr-1(ky13) X; vxEx1175[Pgcy-32::GFP Punc-122::GFP*].

### 4.2 Microscopy and dendrite scoring

For the dendritic scoring assays, worms were synchronized by timed egg laying and then maintained either on the benchtop for 21% oxygen conditions, or in a Modular Incubator Chamber (Billups-Rothenberg) attached to an oxygen tank containing 1% or 10% O_2_ balanced with nitrogen (Airgas). On the day of the assay, either day one or day four adults were mounted on 2% agarose pads, anesthetized with 30-mM sodium azide diluted in NGM, then visualized on an Olympus IX51 inverted microscope equipped with an X-Cite FIRE LED Illuminator (Excelitas Technologies Corp.) and an Olympus UPlanFL N 40X/0.75 NA objective. Epifluorescence images were taken with a Retiga 2000R CCD camera (QImaging) and QCapture Pro 6.0 software.

Dendrites were scored as complex if they had at least one secondary branch that extended ≥5 ​μm from the primary dendritic stalk ending, and were scored as simple otherwise. We found that in some worms one of the URX neurons would be simple while the other would be complex, and a small number of worms expressed the *Pgcy-32::GFP* transgene in only one neuron of the URX pair. Therefore we scored each dendrite individually, giving an n of 2 or 1 per animal. Image analysis was performed using ImageJ (NIH). For Fig. 1D, dendritic branch length was quantified using the segmented line tool in ImageJ. Pictures were analyzed in pairs to ensure consistent start and end points for branch measurements but blinded to age and condition.

Confocal images were captured with a Zeiss LSM 710 microscope equipped with a Plan-Apo 63X (oil); 1.4 NA objective lens and Zen Imaging software. Images were analyzed in ImageJ and are shown as maximum intensity Z-projections.

For SIM imaging, day four adults grown at 20 ​°C were anesthetized in 110 ​mM sodium azide and 20 ​mM levamisole in M9, then mounted with No. 1.5 coverslips on 3% agarose pads with 110 ​mM sodium azide. Imaging was performed on an OMX V4 Blaze microscope (GE Healthcare) equipped with three watercooled PCO.edge sCMOS cameras, a 488 ​nm laser, and a 528/48 emission filter (Omega Optical). Images were acquired with a 60X/1.42 NA Plan Apochromat objective (Olympus) and a final pixel size of 80 ​nm. Z-stacks of ~3–6 ​μm were acquired with a z-step of 125 ​nm and with 15 raw images per plane (three angles with five phases each). Spherical aberration was minimized using immersion oil matching (Hiraoka et al., 1990); generally, oil with a refractive index of 1.524 worked well. Superresolution images were computationally reconstructed from the raw data sets with a channel-specific, measured optical transfer function, and a Wiener filter constant of 0.001 using custom written 3D-SIM reconstruction code (T. Lambert, Harvard Medical School) based on Gustafsson et al. (2008). Images are displayed as maximum intensity Z-projections. Final image layouts were assembled in Adobe Illustrator.

### 4.3 Molecular biology and transgenic strain construction

Constructs to label URX consisted of an 876-bp fragment of the *gcy-32* promoter amplified from genomic DNA, and either worm-optimized GFP amplified from plasmid pPD95.75, or worm-optimized mCherry amplified from plasmid pCFJ90. These fragments were combined by fusion PCR and then subcloned into a pCR-Blunt vector with the Zero Blunt PCR Cloning Kit from ThermoFisher Scientific. Each construct was injected at 20 ​ng/μl along with either *Punc-122::GFP* or pRF4/*rol-6(su1006)* as a co-injection marker.

Mutations were followed in crosses by PCR genotyping and additionally by phenotype when possible.

### 4.4 Calcium imaging

To image day one adults, we picked L4 animals expressing the YC2.60 Ca^2+^ sensor 24 ​h before imaging. To image day four adults, we picked L4s expressing the sensor five days before the assay and moved them to new plates every two days. We found that this was important to ensure we could discriminate between day four adults and their progeny. On the day of the assay 5–10 worms were glued to agarose pads (2% in M9 buffer, 1 ​mM CaCl_2_), using Dermabond tissue adhesive, with their body immersed in OP50 washed off from a seeded plate using M9 buffer. The animals were quickly covered with a PDMS microfluidic chamber and 7% O_2_ pumped into the chamber for 2 ​min before we began imaging, to allow animals to adjust to the new conditions. Neural activity was recorded for 6 ​min with switches in O_2_ concentration every 2 ​min.

Imaging was on an AZ100 microscope (Nikon) bearing a TwinCam adaptor (Cairn Research, UK), two ORCA-Flash4.0 V2 digital cameras (Hamamatsu, Japan), and an AZ Plan Fluor 2 ​× ​lens with 2 ​× ​zoom. Recordings were at 2Hz. Excitation light from a Lambda LS xenon arc lamp (Sutter) was passed through a 438/24 ​nm filter and an FF458-DiO2 dichroic (Semrock). Emitted light was passed to a DC/T510LPXRXT-Uf2 dichroic filter in the TwinCam adaptor cube and then filtered using a 483/32 ​nm filter (CFP), or 542/27 ​nm filter (YFP) before collection on the cameras. Recordings were analyzed using Neuron Analyser, a custom-written Matlab program available at https://github.com/neuronanalyser/neuronanalyser.

## 5 Bordering assays

Bordering assays were performed as previously described (Gross et al., 2014). Worms were synchronized by selecting L4-stage hermaphrodites and letting them grow to either day one or day four adults for the assay. For each assay plate, 40 adult worms of the appropriate age were transferred to 6-cm diameter NGM plates that had been seeded two days prior with 200 ​μl of an overnight OP50 strain bacterial culture. One hour after transfer, the percentage of worms on the bacterial lawn border was calculated.

## Appendix A Supplementary data

Multimedia component 1 Supplemental Figure 1. **Example images of simple dendritic endings in *egl-19(gf)* double mutants.** Representative images of simple morphology in *egl-19(gf);gcy-35* day four adults and *egl-19(gf);cng-1* day four adults, all grown in high oxygen. The *egl-19(gf);gcy-35* double mutant had normal simple endings, while the *egl-19(gf);cng-1* double mutant had abnormal “blobby” endings. Scale bars in images showing full neuron and inset are 10 ​μm and 5 ​μm, respectively. Supplemental Figure 2. **The *npr-1* loss-of-function mutant borders less with age and grows normal dendritic URX endings**. A.) Bordering of day one and day four *npr-1(ky13)* mutant worms. Example image shown on the left. Day four adults were less often found on the border compared to day one adults. N ​= ​12 assays for day one and day four. p ​< ​0.001. B.) Quantification of dendritic ending morphology in day four wild-type and *npr-1(ky13)* mutant worms grown in high oxygen Multimedia component 1
