# Supplementary figures and images for: Long-term activity drives dendritic branch elaboration of a C. elegans sensory neuron
Source: Dev Biol. 2020 May 1;461(1):66–74. doi: 10.1016/j.ydbio.2020.01.005 (PMC7170766; doi:10.1016/j.ydbio.2020.01.005)

*egl-19(gf);gcy-35* day 4 adults - Simple

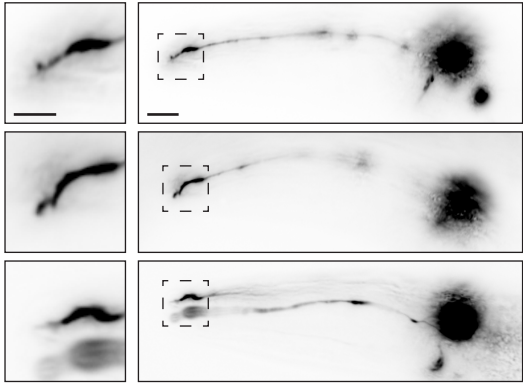

*egl-19(gf);cng-1* day 4 adults - Simple

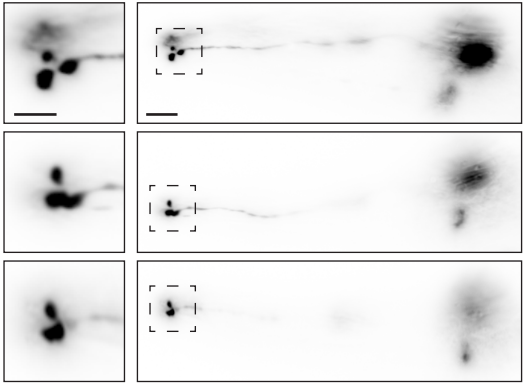

**A**

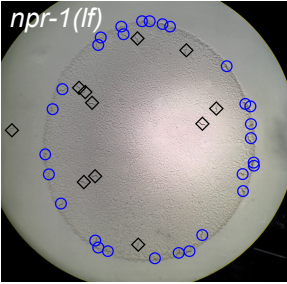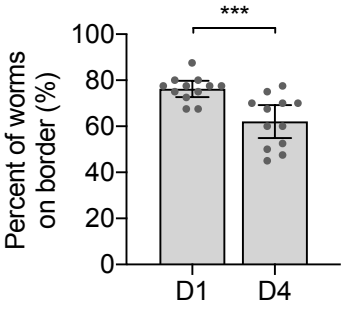

**B**

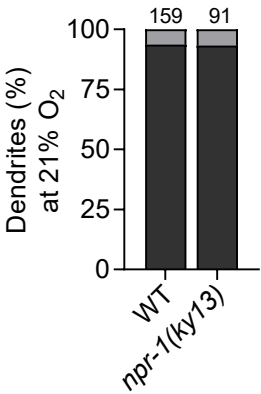

Supplement: Multimedia component 1 — Supplemental Figure 1. Example images of simple dendritic endings in egl-19(gf) double mutants. Representative images of simple morphology in egl-19(gf);gcy-35 day four adults and egl-19(gf);cng-1 day four adults, all grown in high oxygen. The egl-19(gf);gcy-35 double mutant had normal simple endings, while the egl-19(gf);cng-1 double mutant had abnormal “blobby” endings. Scale bars in images showing full neuron and inset are 10 ​μm and 5 ​μm, respectively. Supplemental Figure 2. The npr-1 loss-of-function mutant borders less with age and grows normal dendritic URX endings. A.) Bordering of day one and day four npr-1(ky13) mutant worms. Example image shown on the left. Day four adults were less often found on the border compared to day one adults. N ​= ​12 assays for day one and day four. p ​< ​0.001. B.) Quantification of dendritic ending morphology in day four wild-type and npr-1(ky13) mutant worms grown in high oxygen [file mmc1.pdf]
